# Cardiopulmonary Responses to Sub-Maximal Ergometer Exercise in a Hypo-Gravity Analog Using Head-Down Tilt and Head-Up Tilt

**DOI:** 10.3389/fphys.2019.00720

**Published:** 2019-06-17

**Authors:** Ana Diaz-Artiles, Patricia Navarro Tichell, Francisca Perez

**Affiliations:** ^1^Department of Aerospace Engineering, Texas A&M University, College Station, TX, United States; ^2^Sibley School of Mechanical and Aerospace Engineering, Cornell University, Ithaca, NY, United States

**Keywords:** physiological dose response, artificial gravity, altered gravity, spaceflight countermeasure, head down tilt posture, upright posture

## Abstract

After more than 50 years of spaceflight, we still do not know what is the appropriate range of gravity levels that are required to maintain normal physiological function in humans. This research effort aimed to investigate musculoskeletal, cardiovascular, and pulmonary responses between 0 and 1 g. A human experiment was conducted to investigate acute physiological outcomes to simulated altered-gravity with and without ergometer exercise using a head-down tilt (HDT)/head-up tilt (HUT) paradigm. A custom tilting platform was built to simulate multiple gravitational loads in the head-to-toe direction (Gz) by tilting the bed to the appropriate angle. Gravity levels included: Microgravity (-6°HDT), Moon (0.17g-Gz at +9.5°HUT), Mars (0.38g-Gz at +22.3°HUT), and Earth (1g-Gz at +90° upright). Fourteen healthy subjects performed an exercise protocol at each simulated gravity level that consisted of three work rates (50W, 75W, 100W) while maintaining a constant cycling rate of 90 rpm. Multiple cardiopulmonary variables were gathered, including volume of oxygen uptake (VO_2_), volume of carbon dioxide output (VCO_2_), pulmonary ventilation (V_E_), tidal volume (V_T_), respiratory rate (R_f_), blood pressure, and heart rate (HR) using a portable metabolic system and a brachial blood pressure cuff. Foot forces were also measured continuously during the protocol. Exercise data were analyzed with repeated-measures ANOVA with Bonferroni correction for multiple comparisons, and regression models were fitted to the experimental data to generate dose-response curves as a function of simulated AG-levels and exercise intensity. Posture showed a main effect in all variables except for systolic blood pressure. In particular, VO_2,_ VCO_2_, V_E_, V_T_, R_f_, and HR showed average changes across exercise conditions between Microgravity and 1 g (i.e., per unit of simulated AG) of -97.88 mL/min/g, -95.10 mL/min/g, -3.95 L/min/g, 0.165 L/g, -5.33 breaths/min/g, and 5.05 bpm/g, respectively. In the case of VO_2_, further pairwise comparisons did not show significant differences between conditions, which was consistent with previous studies using supine and HDT postures. For all variables (except HR), comparisons between Mars and Earth conditions were not statistically different, suggesting that ergometer exercise at a gravitational stress comparable to Mars gravity (∼3/8 g) could provide similar physiological stimuli as cycling under 1 g on Earth.

## Introduction

Astronauts on space missions experience various detrimental physiological effects including (but not limited to) muscular atrophy, diminished cardiopulmonary function, and redistribution of internal fluids (Clément, 2005; Buckey, 2006). These changes can lead to orthostatic intolerance (Buckey et al., 1996b; Lee et al., 2015) and diminished exercise capacity (Levine et al., 1996) upon return to a gravitational environment. Currently, crewmembers in the International Space Station (ISS) use a series of countermeasures to attenuate these detrimental effects. For example, astronauts exercise 2.5 h a day (including set-up and cleaning time), 6 days a week, on three different exercise devices: a cycle ergometer, a treadmill, and the advance resistive exercise device (ARED) (Diaz et al., 2015a). Other countermeasures include fluid loading (Clément and Bukley, 2007), and the use of Lower Body Negative Pressure (LBNP) (Charles and Lathers, 1994). Although these countermeasures have greatly diminished the degree of deconditioning experienced during ISS missions (Smith et al., 2012; Moore et al., 2015), especially since the introduction of the ARED, (Smith et al., 2012; Petersen et al., 2016), they still require a significant amount of time and resources. Additionally, the current ISS exercise hardware suite is bulky and most likely, it will not be available in future long duration exploration missions to the Moon, Mars, and beyond due to volume, mass, and power constraints in future exploration vehicles (Ploutz-Snyder et al., 2018). Instead, smaller exercise devices that combine aerobic and resistance exercise capabilities are currently being investigated (Ploutz-Snyder et al., 2018). Thus, new approaches, potentially combining current and novel countermeasures, are likely to be needed for longer space missions in the future, especially on a trip to another planetary surface where there will not be any ground support for astronauts after landing.

Artificial gravity (AG) combined with exercise has been proposed as a multi-system countermeasure that can provide benefits to multiple physiological systems at once (Hargens et al., 2013; Paloski and Charles, 2014; Clément et al., 2016; Clément, 2017). However, the specific parameters and conditions (i.e., gravitational level, intensity, duration) under which this exercise should be ideally performed to be most effective are still unknown. More broadly, there is a lack of fundamental understanding of the relationship between gravity level (i.e., gravitational dose) and physiological response. This relationship, also known as gravitational dose-response curve, will not only contribute to determining physiological responses at Moon and Martian gravity levels, but also the gravity range in which a particular physiological response is closest to “Earth response,” and therefore the range of AG that would most likely be effective as a countermeasure (Clément, 2017).

Previous ground-based investigations on cardiopulmonary responses to ergometer exercise in altered-gravity have focused primarily on studying hypergravity conditions (>1 g), especially through the use of small radius centrifuges equipped with cycle ergometers (Bonjour et al., 2010, 2011; Diaz Artiles, 2015; Diaz et al., 2015b; Diaz Artiles et al., 2016; Diaz-Artiles et al., 2018). When hypogravity conditions (<1 g) have been investigated, most studies have been limited to small experiments in actual microgravity conditions during spaceflight that resulted in very few data points, typically less than four subjects, and they only compared 0 to 1 g conditions (Girardis et al., 1999; Trappe et al., 2006; Bonjour et al., 2011). Other studies conducted simulations on Earth that included the use of Lower Body Positive Pressure (LBPP) (Cutuk, 2006; Evans et al., 2013; Schlabs et al., 2013), parabolic flights (Pletser, 2004; Pletser et al., 2012; Widjaja et al., 2015), and head-down tilt (HDT) and head-up-tilt (HUT) paradigms (Lathers et al., 1990, 1993; Louisy et al., 1994; Pavy-Le Traon et al., 1997; Kostas et al., 2014; Baranov et al., 2016). However, few of these hypogravity simulation experiments have studied the effects of exercise (Richter et al., 2017), and those that did focused on walking and running tasks (Cutuk, 2006; Schlabs et al., 2013; Pavei et al., 2015; Pavei and Minetti, 2016), or anaerobic training (Alessandro et al., 2016).

The objective of our research is to investigate and characterize cardiovascular, pulmonary, and musculoskeletal responses (i.e., foot forces) to ergometer exercise in hypogravity conditions (between 0 and 1 g) to fill the gap between those gravitational levels. We conducted a ground-based study on healthy human subjects using a HDT/HUT paradigm to simulate hypogravity conditions on Earth (Clement et al., 2015). Specifically, we investigated the effects of multiple gravity levels (including Microgravity, Moon, Mars, and Earth) by varying posture and exercise intensities to generate gravitational dose-response curves between simulated 0 and 1 g.

## Experimental Methods

### Subjects and Study Approval

Fourteen healthy subjects (12 males, 2 females) capable of performing 1 h of cardiovascular exercise were selected to participate in the experiment (mean ± SD, age: 23.5 ± 3.5 years; height: 177.6 ± 8.0 cm; weight: 71.9 ± 7.8 kg). Prior to the experiment, subjects were asked to complete a questionnaire designed to identify exclusion criteria such as cardiopulmonary medical conditions, recent musculoskeletal injuries, or medications that could put subjects at risk or bias the results. Subjects were also instructed to avoid exercising and to abstain from drinking caffeine the morning prior to testing. All subjects were informed about their right to withdraw from the experiment at any point and provided written informed consent to participate. The study was approved by Cornell University’s Institutional Review Board for Human Participants (protocol ID #: 1706007254).

### Altered-Gravity Exercise Platform Simulator

**FIGURE 1:**
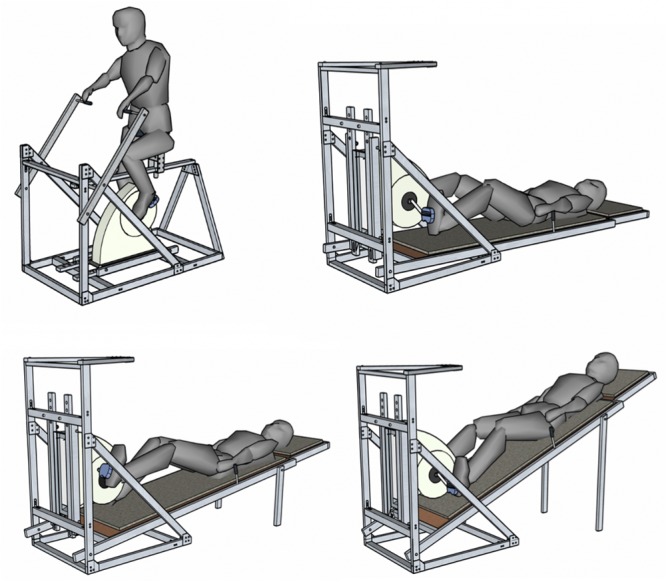
The Altered-gravity Exercise Platform Simulator in upright-Earth configuration (upper left), -6° HDT-Microgravity configuration (upper right), +9.5° HUT-Moon configuration (bottom left), and +22.3° HUT-Mars configuration (bottom right).

The Altered-gravity Exercise Platform Simulator (AEPS) is a custom-built platform designed to perform cycling ergometer exercise in multiple, simulated gravitational environments. Using HDT and HUT positions, the AEPS can replicate known gravity-induced fluid shifts based on appropriate tilt angles. Thus, the platform is capable of providing a -6° HDT, a +9.5° HUT, a +22.3° HUT, and a +90° upright orientation, corresponding to Microgravity, Moon, Mars, and Earth gravitational conditions, respectively (Clement et al., 2015). In the reclined positions (i.e., Microgravity, Moon, and Mars), subjects laid on the platform and handlebars were positioned laterally on either side, to help them avoid sliding down. The handlebars had five different configurations in order to be adjustable for the subjects’ anthropometric needs. In the upright position, subjects sat on a bike seat with handlebars positioned in front of them as on a standard bike. The platform also incorporated an ergometer device (Lode BV, Groningen, Netherlands) for subjects to perform cycling exercise while exposed to the different postures (see Figure 1). If needed, the cycle ergometer was slightly adjusted to accommodate anthropometric differences between subjects.

### Experimental Design

A within-subject experimental design was implemented to determine the effects of artificial gravity (AG) level and exercise work rates (WR) on cardiopulmonary and musculoskeletal responses. The AG levels tested were: Microgravity (-6° HDT), Moon (+9.5° HUT), Mars (+22.3° HUT), and Earth (+90° upright). The exercise intensities tested were: 50, 75, and 100W. Every subject experienced every combination of WR and AG level (i.e., within-subject design). Each subject participated in four sessions scheduled on different days within the same week. The experimental sessions were always performed in the morning approximately at the same time to avoid possible confounding circadian effects that could influence the results. In each of the four sessions, subjects performed the same exercise protocol in a different posture. Earth configuration (i.e., upright) was always tested first in order to allow subjects to get familiar with the exercise protocol and testing equipment. Then, a counterbalanced design was used for the following three test sessions (i.e., Microgravity, Moon, Mars), meaning that subjects experienced these three AG levels in a different order to counteract potential carryover effects.

### Instrumentation and Data Collection

Volume of oxygen uptake (VO_2_, mL/min), volume of carbon dioxide output (VCO_2_, mL/min), pulmonary ventilation (V_E_, L/min), tidal volume (V_T_, ml), and respiratory rate (R_f_, breaths/min) were recorded continuously throughout the experiment sessions using the K4b2 portable gas analyzer (Cosmed, Srl – Italy). Prior to testing, the K4b2 main unit was warmed-up for a minimum of 45 min as instructed in the system manual. The gas analyzer was calibrated before each test using a reference gas mixture (CO_2_: 5%, O_2_: 16%; Cosmed, Srl-Italy) and the turbine was calibrated once a week with a 3000-ml syringe. The Cosmed K4b2 equipment also measured continuous heart rate data (HR) using a Polar Heart Rate monitor. Blood pressure measurements were taken every 2 min during the entire protocol using an automated brachial blood pressure (BP) monitor connected to the cycle ergometer and controlled by the Lode Ergometer Manager, Version 9.4.4 (LEM, 2013, Groningen, Netherlands) software package. Although subjects were supporting themselves via the handlebars, we asked them to relax their arm while blood pressure measurements were being taken. In addition to BP, other exercise parameters such as pedaling cadence and workload intensity were continuously measured and recorded. Additionally, foot-force data were also collected using force-plates (Vernier Software & Technology) mounted on the ergometer pedals. These sensors measured forces between -850 and +3500 N with a resolution of 1.2 N, where positive force values correspond to compression forces and negative force values correspond to traction forces. The force plates were calibrated before each experimental session. Finally, an exit survey was conducted to collect subjective data about the subjects’ experience during the exercise protocol. Questions included comfort and difficulty of exercise using a 10-point Likert scale (Comfort: 1 = very uncomfortable/ unnatural, 10 = very comfortable/ natural; Strenuousness: 1 = easy, 10 = very strenuous), as well as potential causes contributing to them. Subjects were also asked to report any muscle soreness or discomfort resulting from the platform orientation.

### Exercise Protocol

**FIGURE 2:**
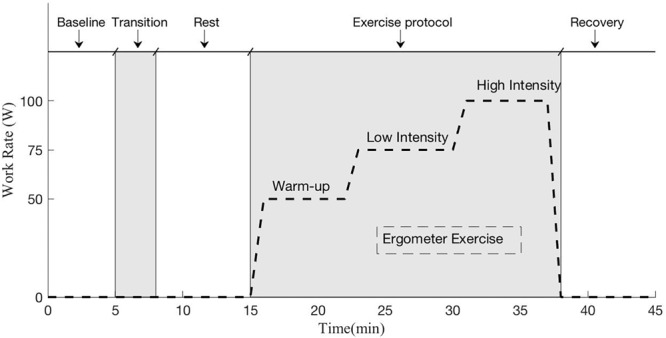
Exercise protocol implemented during each experimental session. The protocol included a baseline session in seated position (5 min), a transition period to position the subject in the desired testing configuration (Microgravity -6° HDT, Moon +9.5° HUT, Mars +22.3° HUT, Earth +90° upright), another period at rest to capture the new baseline in the new gravitational configuration (7 min), the ergometer exercise period (23 min including work rate transitions), and a final recovery period (7 min).

The exercise protocol implemented in all experimental sessions is shown in Figure 2. Each exercise session began with a 5-min resting period in the seated position in order to obtain a physiological baseline at rest. After this first period, subjects were positioned on the platform (or sat on the bike upright for Earth configuration) and were required to rest for seven additional minutes to capture their physiological baseline in the new experimental condition. Subjects then executed the exercise portion of the testing protocol, which consisted of three different workload stages of 50W (“warm-up”), 75W (“low” intensity), and 100W (“high” intensity). All exercise stages were 7-min long. To facilitate transition between work rates and to avoid potential injuries, 30-s transition periods between stages were also included. After the exercise period, an additional 7-min resting period was included to allow subjects to partially recover from the exercise. The exercise protocol was created using the Lode Ergometer Manager, Version 9.4.4 (LEM, 2013, Groningen, Netherlands) software package provided with the ergometer, and it ran automatically without intervention from the operator. Subjects were instructed to pedal at 1.5 Hz (i.e., 90 rpm) using a metronome to avoid additional confounding factors. During the entire protocol subjects were instructed to avoid talking and making unnecessary movements that could affect data collection. Additionally, an early termination protocol was in place to ensure the safety of the subjects throughout the experiment. Termination criteria included an increase in heart rate >0.8^∗^(220- Subject Age), an increase in diastolic blood pressure >20 mmHg with respect to seated baseline measurements, and systolic blood pressure >230 mmHg.

### Data and Statistical Analysis

Breath-by-breath pulmonary variables (VO_2_, VCO_2_, V_E_, V_T_, R_f_) were averaged over 5-s intervals, and outliers were removed using a Hampel filter (Pearson et al., 2016). In addition, a 5th order median filter was also applied to reduce noise of the signals. Each of these variables and the heart rate data (VO_2,_ VCO_2,_ V_E_, V_T_, R_f_, HR), which were collected continuously, were averaged over the last 2 min of each protocol phase, yielding five values per variable, per subject, at each AG configuration. Blood pressure data, which were collected every 2 min, were averaged using the last two values obtained in each protocol phase. Thus, for each posture, we generated averages corresponding to the seated baseline period (BL), at rest (Rest, no exercise in the AG environment of interest), 50, 75, and 100W. To study the effect of postural changes, paired, two-sided *t*-tests were used to compare all cardiopulmonary (CP) variables at BL with Rest at the AG condition being studied. To study the effects of exercise at different postures, a two-way repeated measures ANOVA was implemented using AG-level (Microgravity, Moon, Mars, Earth), and workload intensity (50W, 75W, 100W) as fixed factors. The necessary assumptions including normality, homoscedasticity, and absence of outliers were checked prior to any testing, and the Greenhouse-Geisser correction was applied when the data violated the sphericity assumption (Salkind et al., 2010). Pairwise comparisons were also calculated using the Bonferroni *post hoc* correction.

A quadratic mixed regression model was used to generate dose-response curves between 0 and 1 g for the pulmonary variables measured:

$$y_{\mathrm{ij}}{=\beta }_{0}{+\beta }_{1}^{*}{\mathrm{AG}}^{2}{+\beta }_{2}^{*}{AG+\beta }_{3}^{*}{\mathrm{WR}}^{2}{+\beta }_{4}^{*}{WR+ \beta }_{5}^{*}{\mathrm{AG}}^{*}{WR+u}_{i}{+\varepsilon }_{\mathrm{ij}}$$

where y_ij_ represents the response of the variable measured for the subject *i* (*i* = 1:14) in the condition *j* (*j* = 1:16, combinations of the 4 AG levels and the 4 WR exercise intensities: 0, 50, 75, and 100W). The terms β represent the fixed effects coefficients, with β_0_ being the intercept. The term u_i_ represents the random effects associated with each subject and the within-subject design. When necessary, we used the Akaike Information Criteria (AIC) to select between different regression models (Akaike, 1974). The AIC is a technique for model selection based on information theory that provides a quantitative way to estimate the quality of a model fit. The preferred model is the one that has minimum AIC among all the other models.

Maximum and minimum peak force values for the right and left foot were calculated as the average of the individual maximum and minimum peak forces for each exercise work rate at each of the simulated altered-gravity configuration, following the same methodology reported in a previous publication (Diaz et al., 2015b). Transitions between stages were not included. Data from two subjects were discarded due to problems with the foot sensors and thus, only twelve subjects were considered. A two-way repeated-measures ANOVA and *post hoc* pairwise comparison with Bonferroni correction were conducted to investigate the influence of altered-gravity and workload intensity. AG-levels considered in this study were Microgravity, Moon, and Mars. Earth configuration was not included due to the differences in body position and pedaling strategy between the reclined positions on the platform (i.e., Microgravity, Moon, Mars) and the Earth position, where subjects were seated on a bike saddle. These differences could lead to changes in inertial forces or pedaling effectiveness not caused by changes in the gravitational environment but changes in pedaling configuration, therefore confounding final results. Paired two-sided *t*-tests were used instead to compare Earth results with reclined positions (i.e., Microgravity, Moon, and Mars). Finally, a non-parametric Friedman’s test was implemented to compare the results of comfort and strenuousness in the different postures. All statistical tests were performed with IBM SPSS Statistics 25 software (IBM Corporation) and the significance level was set at α = 0.05.

## Results

All subjects tested were able to successfully complete the exercise protocol except for one subject, who exceeded the maximum blood pressure criteria and thus, the testing session was terminated immediately. The subject was completely excluded from the study (i.e., subject not included in the cohort of 14 subjects analyzed), and therefore any related data have not been included in the results reported in this manuscript.

**FIGURE 3:**
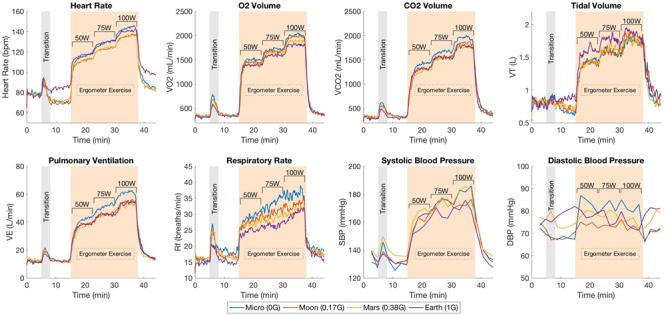
Mean cardiopulmonary responses from 14 subjects during the exercise protocol under the different altered-gravity environments (Microgravity -6° HDT, Moon +9.5° HUT, Mars +22.3° HUT, Earth +90° upright). The protocol includes a baseline period in the upright seated position (5 min), a transition period for the subject to get positioned in the desired testing configuration (Microgravity, Moon, Mars, or Earth), another period at rest to capture the new baseline in the new gravitational configuration (7 min), the exercise phase with three workload intensities (23 min including work rate transitions), and a final recovery period (7 min). Systolic and diastolic blood pressure measurements were collected every 2 min and not continuously as the rest of the variables.

Continuous responses from the cardiopulmonary variables during the different testing configurations are shown in Figure 3. Each graph contains the average cardiopulmonary responses of all 14 subjects (SE not shown for clarity) at each one of the four altered-gravity scenarios investigated (Microgravity -6° HDT, Moon +9.5° HUT, Mars +22.3° HUT, Earth +90° upright). Figure 3 also shows the average systolic and diastolic blood pressure (measurements taken every 2 min) of all 14 subjects during the exercise protocol for all four conditions.

After the 5-min baseline period, a rise in HR can be observed corresponding to the transition between the seated position and the required position according to the altered-gravity level being studied. During the exercise period, HR increased proportionally with the workload intensity as expected. VO2, VCO2 and VE responses showed a similar pattern along the exercise protocol. Abrupt increases can be observed in each transition between different workload levels followed by the establishment of a new relatively steady state in order to meet the new oxygen demand of the body. In contrast, V_T_ and R_f_ showed much more noisy behavior. V_T_ increased after every change in work rate but instead of staying constant, it showed a tendency to decrease. This phenomenon was compensated by the R_f_, which increased during the entire duration of every work rate period. Despite the higher variability, both V_T_ and R_f_ seemed to work closely together to maintain V_E_ at the adequate levels.

### Changes in Posture

**FIGURE 4:**
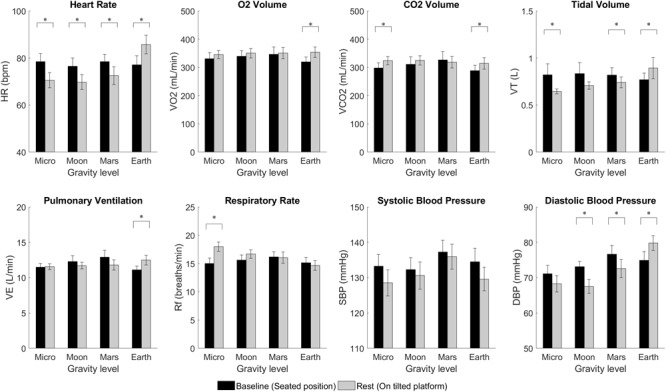
Cardiopulmonary variables from 14 subjects (mean ± SE) at baseline (BL, seated) and at rest (on the platform) for the different altered-gravity environments (Microgravity -6° HDT, Moon +9.5° HUT, Mars +22.3° HUT, Earth +90° upright). The figure highlights significant differences between BL and Rest due to changes in posture (paired *t*-test ^∗^significantly different at *p* < 0.05).

Calculated averages for the six cardiopulmonary variables (VO_2,_ VCO_2,_ V_E_, V_T_, R_f_, HR) and blood pressure measurements (SBP, DBP) for the seated baseline (BL) condition, and at rest (Rest) on the platform at the different simulated altered-gravity environments are summarized in Figure 4. Paired, two-sided *t*-tests revealed the expected significant differences in HR between seated position (BL) and all four postural conditions investigated. Thus, with respect to BL, HR decreased in all hypogravity conditions (Microgravity, Moon, Mars) where the Gz hydrostatic column is reduced, increasing central ventricular filling pressure and ventricular end-diastolic volumes (Nixon et al., 1979; Gaffney et al., 1985). However, it increased in the Earth condition due to the higher gravitational and muscular stresses that result from subjects’ exposure to 1 g while positioned on the exercise platform. VO_2_, VCO_2_, V_T_, V_E_, and DBP also significantly increased with respect to BL in the Earth configuration. Additionally, we observed significant changes in VCO_2_, V_T_, and R_f_ in the Microgravity condition with respect to the seated baseline. With regards to blood pressure, SBP remained fairly constant (i.e., no significant differences were observed) but DBP decreased significantly in Moon and Mars conditions due to vasodilatation, whereas it increased in Earth configuration due to vasoconstriction. DBP also decreased in Microgravity conditions but differences were not statistically significant. For completeness, we also calculated the respiratory exchange ratio (*RER* = *VCO*_*2*_/*VO*_*2*_), reported in Table 1, and paired two-sided *t*-tests results showed no significant differences between BL and Rest in any of the AG conditions.

**Table 1:** Respiratory exchange ratio (**RER=V CO_2_ /V O_2_**) averages [mean (SE), including all 14 subjects] during baseline (BL, seated position), at Rest on the platform, and at each work rate of the exercise protocol (50W, 75W, 100W) at the different simulated altered-gravity positions (Microgravity -6° HDT, Moon +9.5° HUT, Mars +22.3° HUT, Earth +90° upright).

RER	Microgravity -6° HDT	Moon +9.5° HUT	Mars +22.3° HUT	Earth +90° upright
BL	0.91 (0.03)	0.91 (0.03)	0.93 (0.02)	0.90 (0.02)
Rest	0.94 (0.02)	0.93 (0.02)	0.91 (0.02)	0.88 (0.02)
50W	0.96 (0.01)	0.91 (0.01)	0.92 (0.01)	0.95 (0.01)
75W	0.97 (0.01)	0.92 (0.01)	0.93 (0.01)	0.97 (0.01)
100W	0.97 (0.01)	0.93 (0.01)	0.93 (0.01)	0.98 (0.01)

### Altered-Gravity and Exercise

**FIGURE 5:**
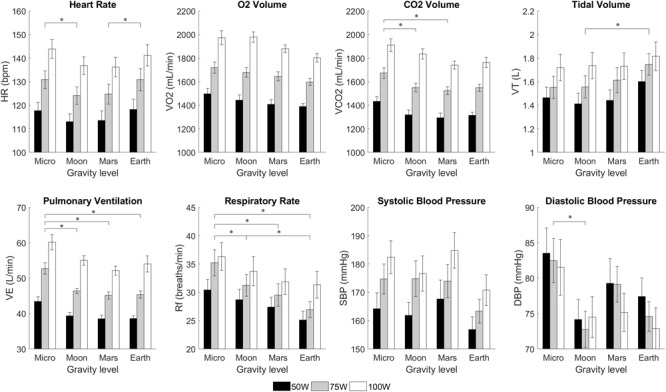
Cardiopulmonary variables from 14 subjects (mean ± SE) at different workload intensities for each of the altered-gravity environments (Microgravity -6° HDT, Moon +9.5° HUT, Mars +22.3° HUT, Earth +90° upright). The figure highlights significant differences between altered-gravity levels (pairwise comparisons after a 2-factor repeated measures ANOVA with Bonferroni correction, ^∗^significantly different at *p* < 0.05). Statistical differences between workload intensities, including all possible pairwise comparisons, were also found in all variables except diastolic blood pressure. For clarity, these differences are not shown in the figure.

Calculated averages for all variables measured during the different workload intensities of the exercise period are shown in Figure 5. Two-way repeated-measures ANOVAs revealed no significant interaction between AG and WR for all variables studied. Further analysis yielded significant main effects of AG-level on all variables except SBP {HR [*F*(1.641,21.337) = 6.148, *p* = 0.011], VO_2_ [*F*(3,39) = 5.838, *p* = 0.002], VCO_2_ [*F*(3,39) = 6.108, *p* = 0.002], V_T_ [*F*(3,39) = 5.546, *p* = 0.003], V_E_ [*F*(3,39) = 10.514, *p* < 0.0005], R_f_ [*F*(3,39) = 15.088, *p* < 0.0005], SBP [*F*(3,27) = 2.315, *p* = 0.098], and DBP [*F*(3,27) = 5.333, *p* = 0.005]}. Thus, our results showed that, when exercising, VO_2_, VCO_2_, V_E_, and R_f_ significantly decreased with higher levels of simulated gravity while V_T_ and HR (except in the Microgravity condition) increased with higher gravitational stress in the Gz direction. Figure 5 also indicates *post hoc* pairwise comparisons, yielding significant differences between Microgravity and Moon on HR, VCO_2_, V_E_, R_f_, and DBP; between Microgravity and Mars on VCO_2_, V_E_, and R_f_; between Moon and Earth on V_T_ and R_f_, and between Microgravity and Earth on V_E_, and R_f_. A similar analysis on RER, shown in Table 1, revealed significant main effects of AG-level {RER [*F*(3,39) = 8.396, *p* < 0.0005]}, followed by significant *post hoc* pairwise comparisons between Microgravity and Moon, and between Microgravity and Mars. No significant differences were found between Mars and Earth conditions except for HR.

Workload intensity was found to be a significant factor in all variables except for DBP {HR [*F*(1.228,15.96) = 281.1, *p* < 0.0005], VO_2_ [*F*(1.169,15.195) = 509.6, *p* < 0.0005], VCO_2_ [*F*(1.402,18.221) = 364.2, *p* < 0.0005], V_T_ [*F*(2,26) = 31.5, *p* < 0.0005], V_E_ [*F*(2,26) = 220.4, *p* < 0.0005], R_f_ [*F*(1.313,17.063) = 24.778, *p* < 0.0005], SBP [*F*(2,18) = 35.495, *p* < 0.0005], and DBP [*F*(2,18) = 0.919, *p* = 0.417]}. For all variables where WR was a significant factor (i.e., all except DBP), WR pairwise comparisons were also found to be statistically significant in all group combinations with no exception. Finally, concerning RER, work rate was found to be a significant factor {RER [*F*(2,26) = 4.936, *p* = 0.015}. However, when tested for pairwise comparisons we did not find significant differences between conditions.

### Dose-Response Curves

**FIGURE 6:**
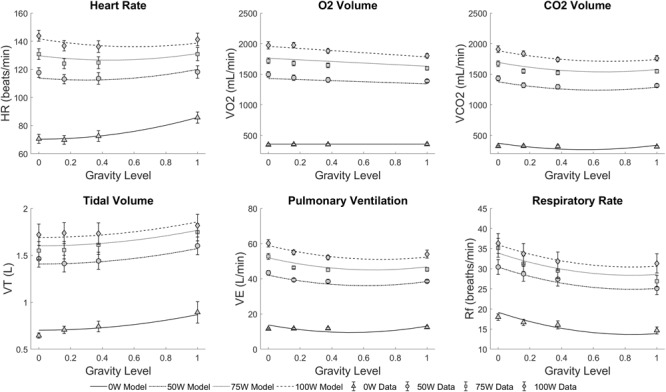
Statistically significant regression models fitted to the cardiopulmonary experimental data across all conditions: four altered-gravity levels (Microgravity -6° HDT, Moon +9.5° HUT, Mars +22.3° HUT, Earth +90° upright) and four workload intensities (0, 50, 75, and 100W). Symbols and error bars correspond to experimental data from 14 subjects (mean ± SE) at each condition.

The regression model coefficients applied to the cardiopulmonary data are provided in Table 2, and the statistical models fitted to the experimental data are shown in Figure 6. Only statistically significant coefficients were included in the regressions, and further interaction terms (not shown) were not significant. Similar to previous gravitational dose-response curves under orthostatic stress generated by short-radius centrifugation (Diaz-Artiles et al., 2018), results show that AG level contributes to changes in all variables, either directly (**β_1_** and β_2_) or through the interaction term (β_5_). Figure 6 shows that generally HR and V_T_ increase with AG, especially between Moon and Earth condition, while VO_2_, VCO_2_, V_E_, and R_f_ decrease with gravity level. As expected, workload intensity also has a prominent role in the regressions, as shown by the significant terms β_3_ and β_4_ in all variables. The positive nature of coefficient β_4_ indicates that all variables increase with workload intensity, and the negative coefficient β_3_ indicates that this increase becomes less important at higher work rates. Systolic and diastolic blood pressure dose-response curves were not included in this analysis due to poor fitting when generating the models. Blood pressure is the “regulated variable” and experimental data did not show a consistent behavior when changing postures as the other variables did. Thus, within the limits of our testing conditions, we were unable to generate appropriate dose-response curves for neither systolic nor diastolic blood pressure.

**Table 2:** Regression coefficients for cardiopulmonary variables based on the following equation: *y*_*ij*_ =β_0_ +$\beta_{1}^{*}$*AG*^*2*^ +$\beta_{2}^{*}$*AG*+$\beta_{3}^{*}$*WR*^2^ +$\beta_{4}^{*}$*WR*+$\beta_{5}^{*}$*AG*^∗^*WR*+*u*_*i*_ +ε_*ij*_.

	β_0_	β_1_	β_2_	β_3_	β_4_	β_5_
HR (bpm)	70.29	15.46	NS	–0.003	1.025	–0.185
VO_2_ (ml/min)	356.59	NS	NS	–0.109	26.93	–1.74
VCO_2_ (ml/min)	370.78	347.71	–380.93	–0.099	25.07	–1.10
T_V_ (l)	0.702	0.165	NS	–8.42 × 10^-5^	0.018	NS
V_E_ (l/min)	13.72	15.63	–16.27	–0.002	0.680	–0.059
R_f_ (min^-1^)	19.17	7.74	–13.07	–0.001	0.282	NS

*Only significant coefficients were included in the regression (*p* < 0.05). Figure 6 shows the regression models fitted to the experimental data. Abbreviations: NS, non-significant; AG, artificial gravity (g); WR, work rate (W); HR, heart rate (beats per minute); VO_*2,*_ volume of oxygen uptake (mL/min); VCO_*2,*_ volume of carbon dioxide output (mL/min); V_*E*_, pulmonary ventilation (L/min); V_*T,*_ tidal volume (mL); R_*f*_, respiratory rate (breaths/min).*

### Foot Forces

Calculated averages for minimum and maximum right and left foot forces during the different workload intensities of the exercise period at the different simulated altered-gravity environments are shown in Table 3. No significant differences were found between right and left foot forces (between subjects’ effect two-way repeated measures ANOVA; for maximum foot forces: *F*(1,22) = 0.008, *p* = 0.927; for minimum foot forces: *F*(1,22) = 0.269, *p* = 0.609). Consequently, both feet were analyzed together.

**Table 3:** Calculated minimum and maximum foot force averages [mean (SE), including 12 subjects] during each work rate of the exercise protocol (50W, 75W, 100W) at the different simulated altered-gravity positions (Microgravity -6° HDT, Moon +9.5° HUT, Mars +22.3° HUT, Earth +90° upright).

		Right Foot (N)		Left Foot (N)	
		Maximum	Minimum	Maximum	Minimum
Microgravity -6° HDT	50W	89.63 (6.71)	–1.49 (7.23)	94.94 (7.64)	–0.11 (5.78)
	75W	94.66 (6.77)	–5.16 (8.32)	98.44 (7.72)	–7.30 (5.60)
	100W	105.42 (6.59)	–6.42 (10.26)	106.46 (6.90)	–6.91 (6.48)
Moon +9.5° HUT	50W	125.36 (5.71)	28.90 (4.72)	125.12 (6.42)	23.75 (4.70)
	75W	141.08 (6.14)	26.84 (5.58)	136.54 (7.07)	18.18 (4.65)
	100W	150.44 (7.54)	22.79 (6.72)	144.74 (8.19)	16.88 (4.54)
Mars +22.3° HUT	50W	153.07 (8.64)	41.77 (6.52)	149.60 (9.64)	36.89 (4.70)
	75W	162.27 (9.33)	34.88 (8.05)	160.61 (10.66)	29.31 (6.68)
	100W	170.19 (8.05)	31.54 (8.65)	167.21 (10.39)	27.67 (6.67)
Earth +90° upright	50W	147.61 (8.24)	53.09 (6.01)	152.44 (10.71)	49.50 (4.69)
	75W	167.33 (8.88)	51.63 (7.20)	169.96 (11.47)	46.67 (6.12)
	100W	185.42 (9.01)	55.22 (7.23)	186.96 (10.63)	53.20 (5.24)

**FIGURE 7:**
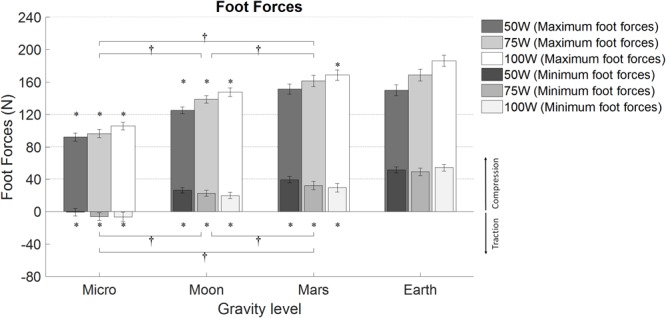
Average minimum and maximum foot forces (12 subjects, mean ± SE at different workload intensities for each of the altered-gravity environments (Microgravity 6° HDT, Moon 9.5° HUT, Mars 22.3° HUT, Earth upright). The figure highlights significant differences between altered-gravity conditions Microgravity, Moon, and Mars (pairwise comparisons after a 2-factor repeated measures ANOVA with Bonferroni correction, † significantly different at *p* < 0.05). Additionally, significant differences compared to Earth are also included (^∗^paired *t*-test, *p* < 0.05). For clarity, pairwise comparisons between workload levels are not shown.

Maximum foot forces were all positive and therefore compression forces, and they are indicated in Figure 7. Statistical analysis showed a significant increase with workload intensity [*F*(1.288, 29.625) = 46.534, *p* < 0.0005] and simulated altered-gravity [*F*(1.583, 36.405) = 204.137, *p* < 0.0055]. Pairwise comparisons revealed significant differences between all work rates (*p* < 0.0005) and all altered-gravity conditions (*p* < 0.0005). Additionally, when compared to Earth’s values (paired, two-sided *t*-test), maximum foot forces were statistically different in Microgravity (all work rates, *p* < 0.0005), Moon (all work rates *p* < 0.0005), and Mars at 100W (*p* < 0.014).

Minimum foot forces were both negative (in the Microgravity configuration) and positive (in the rest of configurations). Thus, minimum foot forces in Microgravity were traction forces, while in the rest of configurations they were compression forces (see Figure 7). A two-way repeated-measures ANOVA showed a significant increase in minimum foot forces with altered-gravity [*F*(1.407, 34.045) = 45.985, *p* < 0.0005] and a significant decrease with workload intensity [*F*(1.443, 33.19) = 16.055, *p* < 0.0005]. Pairwise comparisons yielded statistically significant differences between all altered-gravity conditions: Microgravity and Moon (*p* < 0.0005), Microgravity and Mars (*p* < 0.0005), and Moon and Mars (*p* = 0.007). Work rate pairwise comparisons showed statistically significant differences between 50W and 75W (*p* < 0.005), and 50W and 100W (*p* < 0.0005). Additionally, when compared to Earth’s values (paired, two-sided *t*-test), minimum foot forces were statistically different in Microgravity (all work rates *p* < 0.0005), Moon (all work rates *p* < 0.0005), and Mars (50W: *p* = 0.004; 75W and 100W *p* < 0.0005).

### Subjective Data

Subjective data related to “comfort” and “strenuousness” (or difficulty of exercise) are summarized in Table 4. Results show a significant increase in comfort level with increased AG [χ^2^ (3) = 23.59, *p* < 0.0005]. Earth was reported as the most natural position with few discomfort issues, mainly related with the bike saddle. In reclined configurations, main causes of discomfort were pressure in the lower back and use of handlebars to avoid sliding. Some cases of numbness in the feet were also reported in the Microgravity configuration, probably due to the head-down tilt of the body and the upper position of the legs. The Friedman test also yielded a statistically significant effect of AG on strenuousness [χ^2^(3) = 27.51, *p* < 0.0005]. Thus, Microgravity was the most challenging position, and the perception of difficulty of exercise was progressively reduced with increasing AG. Subjects were also asked to choose between workload intensity and protocol duration as the main cause of strenuousness. Workload intensity was selected in the majority of the cases. Other factors such as cycling frequency and position discomfort were also mentioned.

**Table 4:** Exit survey results concerning perceived comfort and strenuousness during the experimental sessions [mean (SE), including 14 subjects] for all altered-gravity conditions (Microgravity -6° HDT, Moon +9.5° HUT, Mars +22.3° HUT, Earth +90° upright).

Ag level	Comfort	Strenuousness
Microgravity -6° HDT	4.4 (2.0)	6.1 (2.1)
Moon +9.5° HUT	5.9 (1.5)	4.9 (2.0)
Mars +22.3° HUT	6.7 (2.2)	4.2 (1.6)
Earth +90° upright	7.4 (2.0)	3.4 (1.7)

*Data was collected using a 10-point Likert scale (Comfort: 1 – very uncomfortable/unnatural, 10 – very comfortable/natural. Strenuousness: 1 – easy, 10 – very strenuous).*

## Discussion

This study measured cardiopulmonary responses to submaximal ergometer exercise under multiple postural conditions using a HDT/HUT paradigm. Although a few studies have reported cardiorespiratory responses to exercise in upright, supine, and -6° HDT, this is the first study to include additional tilt angles representing Moon and Mars gravitational conditions, which allowed the generation of additional data points to build cardiopulmonary dose-response curves in simulated hypogravity at multiple exercise intensities.

Head-down tilt posture at an angle of 6° has become the standard model for microgravity simulation (Pavy-Le Traon et al., 2007). In this position, fluids are redistributed toward the central cavity, causing an increase in ventricular preload, left-ventricular end-diastolic volume, and stroke volume compared to upright and also supine posture. It is important to note that in -6° HDT, the Gx hydrostatic gradient and a small Gz (foot-to-head) hydrostatic gradient exist, making this condition slightly different from supine posture (where Gz gradient does not exist), and different from true microgravity conditions (no hydrostatic gradients exist).

Cardiopulmonary responses at rest due to postural changes are consistent with previous studies. Prisk et al. (1995) measured resting pulmonary gas exchange in eight subjects in standing upright, supine, and microgravity conditions (Spacelab flights SLS-1 and SLS-2). Their results showed that V_T_ decreased in the supine position with respect to standing upright, and it decreased even more in real microgravity conditions. As a compensatory mechanism, they noted an increase in R_f_ when subjects were in microgravity (but not in supine). In our experiment, we found a significant decrease in V_T_ and a significant increase in R_f_ when transitioning from seated upright to -6° HDT condition, which is consistent with previous results given that our testing conditions differ slightly likely creating a larger perturbation to the pulmonary system. Our results also support the idea that changes in the gravitational conditions and thus hydrostatic pressures cause subjects to select a different combination of V_T_ and R_f_ to maintain the appropriate alveolar ventilation (Prisk et al., 1995). Given the adjustments in V_T_ and R_f_, our subjects were able to also maintain a similar V_E_, contrary to Prisk’s study where subjects experienced a significant decrease in V_E_ both in supine and microgravity. Our V_T_ and R_f_ results are also consistent with ventilatory responses gathered by Gisolf et al. (2004) on eight normal subjects in supine and standing position. With respect to gas exchange, we also found unaltered VO_2_ and slightly elevated VCO_2_ in -6° HDT compared with sitting upright. When comparing the upright seated baseline with the Earth condition baseline (i.e., subjects are seated on the bike saddle ready to start pedaling), we did see significant increases in VO_2_, VCO_2_. V_T_, and V_E_. This is consistent with subjects not being truly in a resting position similar to the other testing conditions (i.e., Microgravity, Mars, Moon) where subjects are laying on top of the tilt platform without exerting any effort to maintain themselves upright (Piotrowski et al., 2018).

Concerning cardiopulmonary responses during exercise, we found a slight tendency of VO_2_ to decrease with increasing gravity levels (2-way repeated measures ANOVA). The average reduction across exercise conditions of VO_2_ per unit of simulated AG-level was -97.88 mL/min/g. This slight reduction is also supported by a reduction in VCO_2_ with increasing gravity levels (-95.10 mL/min/g averaged across exercise conditions). These changes in cardiopulmonary variables may be related to the suggestion that exercise performance decreases with reducing hydrostatic column (Egaña et al., 2006). The exact mechanisms may be attributed to a decrease in muscle perfusion at higher tilt angles with respect to upright (Fitzpatrick et al., 1996; Wright et al., 1999), potentially reducing blood flow to the exercising muscles (Nielsen, 1983), and an attenuated muscle pump effect in the presence of a reduced (or even absence in the case of HDT) venous hydrostatic column (Laughlin, 1987; Laughlin and Joyner, 2003). However, we did not find significant differences between conditions in VO_2_ when tested for pairwise comparisons, indicating that if this effect exists, it does not seem to be very strong. This is consistent with previous results collected during submaximal cycling exercise at 100W in both upright and -6° HDT conditions reporting no significant differences in VO_2_ or HR between the two postures, either before or after an 8-week training protocol (Ade et al., 2013). Also consistent with Ade et al. (2013), our HR data at 100W in upright (Earth condition), and -6° HDT (Microgravity condition) do not differ either. However, it is interesting to note that HR does decrease in intermediate postures (Moon and Mars conditions). This non-linear behavior could be related to differences between HUT and HDT and the reversal of the hydrostatic gradient along the Gz- body axis, as well as the discomfort and additional effort reported by our subjects associated to cycling during HDT when the legs are elevated above heart level (Ade et al., 2013).

Cardiopulmonary data on true microgravity during ergometer exercise are very scarce. One study was conducted onboard the Russian Space Station Mir (Girardis et al., 1999) to investigate metabolic consumption of two astronauts during cycle exercise at 50, 75, and 100W. Results showed that submaximal VO_2_ at 0 g was significantly lower than all measurements taken at 1 g. These results are in disagreement with our ground-study as well as with results reported by others during HDT on Earth (Ade et al., 2013). This discrepancy between spaceflight and supine and/or HDT data raises again the question about the validity of -6° HDT as a good microgravity analog when studying submaximal exercise capacity. Other factors to take into account are that spaceflight data were collected in only two subjects, and the first data points were taken on flight day 12, and thus potential effects of muscle and aerobic capacity deconditioning might have occurred by then. Additionally, atmosphere composition at the time of the test (which was not reported) as well as pedaling frequency (which was not controlled by an operator) might have been additional factors affecting the results. All in all, further studies in true microgravity (and ideally true partial gravity) are warranted to better elucidate submaximal exercise pulmonary responses in these conditions.

Using these spaceflight data as well as additional hypergravity data, Bonjour et al. (2011) developed a model to predict cardio-pulmonary responses to submaximal cycling exercise in varying gravitational environments. Gravitational levels included 0 g (data from MIR reported above), as well as hypergravity data collected on centrifuges in Karolinska Institute in Sweden (1, 1.5, 2, and 2.5 g), and the Center for Research and Education in Special Environments in Buffalo (1, 2, and 3 g). Bonjour proposed a quadratic non-linear relationship between HR and AG, and he predicted HR responses at Mars and Moon gravity levels when cycling at 50, 75, and 100W. The comparison between his predictions and the results obtained in this study is shown in Table 5. When analyzing the responses at Earth condition (1 g), we can observe that our results showed higher HR responses than the ones obtained by Bonjour. Individual differences between the subjects could likely explain this effect, suggesting that the subjects participating in our study were less physically prepared than the ones participating in the studies he conducted. Another important factor is the difference in pedaling frequency. Bonjour considered studies performed at a pedaling frequency of 1 Hz whereas our study was conducted at 1.5 Hz. This difference leads to an increased cycling internal work (Bonjour et al., 2010), which makes our study more physically demanding.

**Table 5:** Comparison between Bonjour’s predictions of the heart rate responses to exercise on Moon and Mars with the results obtained in this study [mean (SE), including 14 subjects].

HR (beats/min)		50W	75W	100W
Moon	Prediction	87	96	105
	AEPS study	113.08 (3.31)	124.09 (3.66)	136.80 (3.75)
Mars	Prediction	88	97	106
	AEPS study	113.51 (4.14)	124.71 (4.16)	136.21 (4.14)
Earth	Prediction	93	102	112
	AEPS study	118.18 (4.44)	130.86 (4.74)	141.21 (4.55)

During exercise, our results show a slight decrease in V_T_, and an increase in Rf with lower tilt angles (i.e., lower simulated gravity levels). Averaged changes with simulated AG-level for V_T_ and Rf across exercise conditions were 0.165 L/g and -5.33 breaths/min/g, respectively. Changes in V_T_ are presumably related to the increase in thoracic pressure when being reclined (Gisolf et al., 2004). The decrease in the tilt angle reduces the gravity force in the longitudinal axis (Gz) but increases it in the direction perpendicular to the platform (i.e., Gx, the anterioposterior direction). As a consequence, there is a higher contribution of the weight forces of the thoracic cage, thus hampering lung expansion. To counteract for this effect, Rf increases at lower tilt-angles, causing also an increase in V_E_. Finally, we did not find any significant changes in blood pressure, except of DBP in simulated microgravity conditions. Diastolic blood pressure significantly increased while exercising at -6° HDT, most likely driven by the introduction of the foot-to-head hydrostatic gradient, combined with the higher positioning of the exercising legs above the Gz line of the body. It is interesting to note that we did not find statistically significant differences between Mars and Earth in any of our cardiopulmonary variables, except for HR.

Previous studies performed onboard the ISS using the Cycle Ergometer with Vibration Isolation System (CEVIS) device measured foot forces between 7.0 and 19.0% of the body weight (BW) using work rates ranging from 75 to 210W (Genc et al., 2010). These results are in concordance with our results in -6° HDT where we obtained maximum peak forces between 12.7 and 14.6% BW. Other studies compared Earth with microgravity cycling loadings, obtaining a 20% and 10% BW, respectively (Cavanagh et al., 2010). Although these results are slightly lower compared to the ones obtained in our study, differences in pedaling rate or workload level can produce changes in foot forces, this being a possible reason for these differences. Foot forces during cycling activity at partial-gravity such as the Martian or the lunar gravitational environments have not been previously studied. However, ground reaction forces at reduced gravity levels during other exercise modalities have been investigated, including running (Cavanagh et al., 2017), hopping (Weber et al., 2019), and the use of stair-steppers (Edmonds et al., 2008). However, ergometer exercise does not provide foot forces as high as these other exercise modalities, and therefore, a straight comparison across different exercise disciplines is not directly applicable. Finally, it is interesting to note that, in our study on ergometer exercise, we did not find considerable differences in maximum foot forces between Mars and Earth conditions. Investigating this similarity could be key with regard to prescription exercise protocols during future long duration exploration missions.

Subjective data in simulated microgravity conditions showed the lowest comfort punctuation. Major complaints reported were lower back pain and discomfort in the arms due to the handlebars. Future suggestions that could improve these issues include a better platform lining, intermediate cycle ergometer positions, and different handlebar configurations to enable a more ergonomic positioning of the subject on the platform. However, no cycling difficulties or major discomfort problems were reported, validating the correct functioning of the platform. Strenuousness results showed an increased difficulty perception at lower artificial gravity levels, which was likely related to the more unnatural positioning of the body.

### Limitations

We used a HDT/HUT paradigm to study the effect of postural changes during submaximal aerobic exercise. -6 °HDT has been adopted by the spaceflight community as a well-accepted analog to simulate microgravity, especially with regard to fluid shifts and cardiovascular adaptations. However, this is not a fully accurate ground-based simulation of spaceflight. The presence of a small longitudinal (Gz, foot-to-head) and transverse (Gx) gravitational effects, including the pressure of the ground’s surface on the subject’s back and their impact on intrathoracic volume and ventilation mechanics, is certainly a limitation when comparing pulmonary and cardiovascular function with true microgravity or true partial gravity (Regnard et al., 2001; Watenpaugh, 2016). A classic example of differences in cardiovascular responses between HDT and true microgravity is the “surprising” reduction of central venous pressure (CVP) in space relative to 1 g supine and upright values, while ground-based analogs such as HDT produce the “expected” increase in CVP related to the central fluid shift (Buckey et al., 1996a; Watenpaugh, 2016). Subjects also reported some discomfort during the exercise sessions in tilted positions, especially the HDT configuration, that should not occur in real microgravity and might have slightly affected the results. Despite these discrepancies, the tilt paradigm reproduced many of the physiological responses reasonably well (Watenpaugh, 2016), but it is important to acknowledge these differences when interpreting the results. In our study, we investigated the effects of gravity alterations through postural changes on Earth in an attempt to elucidate physiological responses in altered-gravity environments, but further studies in true hypogravity are warranted to fully capture those responses.

We included Earth configuration in our study since 1 g is one of the most interesting conditions when investigating gravitational physiology. However, we acknowledge some differences in configuration and body posture with respect to the reclined positions, such as the presence of a saddle and the need for subjects to keep themselves upright. Thus, a consequence of exercising while lying on the platform is the unloading of the back and trunk muscles, which might decrease the VO_2_ requirements during exercise in these hypogravity configurations (Piotrowski et al., 2018). Given all these differences, subjects always experienced the upright configuration first (could be considered as the familiarization session) and was not included in the randomization scheme implemented with the rest of the sessions.

Other limitations of the study are related to the resources available to conduct the experiment. Subjects were selected from the college population and thus, their age (range between 20 and 32 years old) and most likely physical fitness (although capable of conducting aerobic exercise for an hour), were not truly representative of the astronaut population. Only two women participated in the study and therefore it is not possible to conduct a comprehensive analysis on gender effects. Additionally, the duration of the test sessions was limited to avoid excessive fatigue of subjects during the exercise protocol. Thus, some variables did not reach steady state and extrapolation of results outside the timeframes investigated should be done with caution.

## Conclusion

For the first time, we characterized cardiopulmonary and musculoskeletal responses to submaximal ergometer exercise during postural changes using a HDT/HUT paradigm. We investigated multiple simulated gravity levels (i.e., tilt angles), and exercise intensities, generating dose-response curves to inform future trade-offs and decisions regarding the effects of hydrostatic changes and altered-gravity on human performance. Our results showed that there are not significant differences in human cardiopulmonary and musculoskeletal responses between Mars and Earth experimental conditions. This indicates some degree of similarity in human performance during ergometer exercise under Martian and terrestrial gravitational environments, suggesting that this type of exercise conducted under a gravitational stress of ∼3/8 g could provide similar physiological stimuli than cycling under 1 g on Earth.

We also found some differences between -6° HDT and the (scarce) true microgravity data gathered during spaceflight, highlighting the limitations of using ground-based models to study the complex physiological processes that occur in true microgravity conditions, as well as the need of collecting additional flight data.

## Ethics Statement

All subjects were informed about their right to withdraw from the experiment at any point and provided written informed consent to participate. The study was approved by Cornell University’s Institutional Review Board for Human Participants.

## Author Contributions

AD-A served as an overall supervisor of the research project and provided advice regarding the experimental design, data analysis, and statistics, and she was the primary contributor to the manuscript. PNT assisted in the experimental design and conducted the human experiments, completed the statistical analysis, and contributed to the manuscript. FP contributed to the experimental design and execution of the experiment, and drafting of the manuscript. All authors approved the final manuscript.

## Conflict of Interest Statement

The authors declare that the research was conducted in the absence of any commercial or financial relationships that could be construed as a potential conflict of interest.
